# Supplementary material for: A simple and low-cost electrode based on Nafion-stabilized silver nanoparticles supported on FTO for the electrochemical determination of Pb (II) and Cu (II)
Source: PLoS One. 2025 Apr 24;20(4):e0320227. doi: 10.1371/journal.pone.0320227 (PMC12021286; doi:10.1371/journal.pone.0320227)
Supplement: S1 Table — The ICP-MS analysis was performed by SGS del Perú S.A.C. The results were reported in the test report MA2411825 Rev. 0, which included the analysis of 49 metals, including Cu and Pb, from four acid mine drainage samples, using the EPA Method 200.8. The analyzed samples were labeled as M3-2 (Sample 1), and M4-1 (Sample 2). The results for samples 1 and 2 are presented, where copper and lead levels were detected in Sample 1, and only copper in Sample 2, using the AgNPs/Nf/FTO electrode (PDF). [file pone.0320227.s001.pdf]

Supporting Information

A simple and low-cost electrode based on Nafion-stabilized silver nanoparticles supported on FTO for the electrochemical determination of Pb (II) and Cu (II)

Leonardo J. Monroy-Cruz[1], Akemi Morales-Kato[1], Yndira Dolores-Maldonado[1], Katiuska Castro[1], Alen Zimic-Sheen[1], Belén Balta[1], Geraldine J. Otayza-Melgarejo[1], Raúl León[2], Patricia Sheen[1], Wilner Valenzuela[1,3*], Mirko Zimic[1*]

[1] Laboratorio de Bioinformática, Biología Molecular y Desarrollos Tecnológicos. Laboratorios de Investigación y Desarrollo. Facultad de Ciencias e Ingeniería. Universidad Peruana Cayetano Heredia. Lima, Perú.

[2] Laboratorio de Metalurgia y Ciencias de Materiales, NDT Innovations, Inc. Lima, Perú.

[3] Grupo de Investigación Electroquímica Aplicada. Facultad de Ciencias. Universidad Nacional de Ingeniería. Lima, Perú.

* Corresponding authors:

E-mail: mirko.zimic@upch.pe (MZ)

E-mail: wvalenzuelab@uni.pe (WV)

Table S1: Analysis Inductively coupled plasma mass spectrometry (ICP-MS). The ICP-MS analysis was performed by SGS del Perú S.A.C. The results were reported in the test report MA2411825 Rev. 0, which included the analysis of 49 metals, including Cu and Pb, from four acid mine drainage samples, using the EPA Method 200.8. The analyzed samples were labeled as M3-2 (Sample 1), and M4-1 (Sample 2). The results for samples 1 and 2 are presented, where copper and lead levels were detected in Sample 1, and only copper in Sample 2, using the AgNPs/Nf/FTO electrode.

Element	LD (mg/L)	LC (mg/L)	M3-2 (Sample 1) (mg/L)	M4-1 (Sample 2) (mg/L)
**Total Copper**	0.00003	0.00009	1.52823 ± 0.38	4.07952 ± 1.020
**Total Lead**	0.0002	0.0006	0.0830±0.0075	0.0020±0.00020
**Total Chromium**	0.0001	0.0003	0.1201 ± 0.030	0.0333 ± 0.0083
**Total Iron**	0.0004	0.0013	375.6347 ± 30.05	3,456.9240 ± 276.55
**Total Zinc**	0.0008	0.0026	62.4271 ± 6.24	798.9679 ± 79.90
**Total Aluminum**	0.001	0.003	17.634 ± 1.59	26.326 ± 2.37
**Total Arsenic**	0.00003	0.00010	1.70679 ± 0.19	1.49006 ± 0.16
**Total Cadmium**	0.00001	0.00003	0.17346 ± 0.040	0.53134 ± 0.12

*LD: Limit of Detection

**LC: Limit of Quantification
