# Supplementary material for: A simple and low-cost electrode based on Nafion-stabilized silver nanoparticles supported on FTO for the electrochemical determination of Pb (II) and Cu (II)
Source: PLoS One. 2025 Apr 24;20(4):e0320227. doi: 10.1371/journal.pone.0320227 (PMC12021286; doi:10.1371/journal.pone.0320227)
Supplement: Attachment — Submitted filename: PLOS-One review.docx [file pone.0320227.s002.docx]

Review report of manuscript (PONE-D-24-33828) entitled “ A simple and Low-cost Electrode Based on Nation-stabilized Silver Nanoparticles Supported on FTO for the Electrochemical Determination of Pb (II) and Cu (II)

Decision: Major revision

This manuscript emphasizes on the detection of heavy metals like Pb and Cu in water samples using electrochemical sensor based on a fluorine-doped tin oxide (FTO) electrode modified with silver nanoparticles (AgNPs) and stabilized with Nafion. The reported detection limits for Pb (II) and Cu(II) are 8.87 ppb and 3.26 ppb. Based on the results and interpretations reported, a major revision is recommended for the present manuscript. The comments are mentioned below.

1. Authors have reported that sensor is selective and sensitive for Pb (II) and Cu (II). However, no other heavy elements are analyzed which poses a question on the selectivity of the sensor. Moreover, a selective sensor will have high response to one analyte than the others investigated so claiming sensor selectivity for Pb(II) and Cu (II) needs an explanation. Please explain it.

**Abstract**

2. Lines 35-39 need to be rephrased for reader’s convenience.
3. Mention the size of Ag nanoparticles.
4. **Keywords**: Determination is not a key word. Please delete it. Sensor could be replaced by electrochemical sensor

**Introduction**:

5. Mention the concentration levels of Pb and Cu at which they are dangerous for human health.
6. Authors have mentioned only two techniques, AAS and ICP-MS for the detection of heavy metals. It is recommended to include more techniques and emphasize on why reported work is better than existing techniques

**Materials and methods**:

7. Line 186: There should be space between 0.5 and cm^2^.
8. Line 195: Why there is a sign of _ after CV?
9. Line 223-224: The analysis of DPV……standard solutions. Please mention relevant citations.

**Results**

10. Line 245: Caption of Fig.1: What authors mean by FTO-glass nude? Nude is not a proper word. Do authors mean pristine/unmodified? This comment is also for caption of fig.2 (line 257)
11. Line 251: Mention the size of nano particles.
12. Line 257: Caption should be SEM micrographs of ……Authors may mentione the magnification and working voltage in the experimental section.
13. Line 262-265: Nothing is mentioned for fig. 3B and 3C. If required, authors should renumber the figures
14. Line 271: There should be space between 60 and ppb. Same is for line 298
