# Supplementary material for: A simple and low-cost electrode based on Nafion-stabilized silver nanoparticles supported on FTO for the electrochemical determination of Pb (II) and Cu (II)
Source: PLoS One. 2025 Apr 24;20(4):e0320227. doi: 10.1371/journal.pone.0320227 (PMC12021286; doi:10.1371/journal.pone.0320227)
Supplement: Attachment — Submitted filename: Response to Reviewers.docx [file pone.0320227.s004.docx]

Dear Reviewers,

Thank you very much in advance for your review and suggestions, and for making us to improve our manuscript. We have reviewed your comments, and we appreciate each one, as they allow us to improve the manuscript titled, “A Simple and Low-cost Electrode Based on Nafion-stabilized Silver Nanoparticles Supported on FTO for the Electrochemical Determination of Pb(II) and Cu(II).” Below, we detail our responses to the comments and observations made by each of you.

1. **Please ensure that your manuscript meets PLOS ONE's style requirements, including those for file naming. The PLOS ONE style templates can be found at:** [**https://journals.plos.org/plosone/s/file?id=wjVg/PLOSOne_formatting_sample_main_body.pdf**](https://journals.plos.org/plosone/s/file?id=wjVg/PLOSOne_formatting_sample_main_body.pdf) **and** [**https://journals.plos.org/plosone/s/file?id=ba62/PLOSOne_formatting_sample_title_authors_affiliations.pdf**](https://journals.plos.org/plosone/s/file?id=ba62/PLOSOne_formatting_sample_title_authors_affiliations.pdf)

The format of the title page was corrected as per the journal's specifications.

2. **In the online submission form, you indicated that "The data will be available upon request to the corresponding author." All PLOS journals now require all data underlying the findings described in their manuscript to be freely available to other researchers, either 1. In a public repository, 2. Within the manuscript itself, or 3. Uploaded as supplementary information. This policy applies to all data except where public deposition would breach compliance with the protocol approved by your research ethics board. If your data cannot be made publicly available for ethical or legal reasons (e.g., public availability would compromise patient privacy), please explain your reasons on resubmission and your exemption request will be escalated for approval.**

All information, including protocols, analytical assays, and the results of the services provided, has been included within the text and as supplementary material.

3. **Please ensure that you refer to Figure 5 in your text as, if accepted, production will need this reference to link the reader to the figure.**

Figure 5 was renumbered as Figure 6 and referenced in the text.

4. **The paper provides a comparison between ICP-MS and DPV-AgNPs/Nf/FTO, but the comparison is rather limited in terms of concentration range and data points. Specifically, for Cu, only two concentrations are tested, and for Pb, only one. This is insufficient to make a robust comparison, especially given the claim that ICP-MS offers “low detection and quantification limits, high precision, and accuracy.” It is essential to extend the comparison to lower concentration regimes to adequately test the capabilities of the DPV-AgNPs/Nf/FTO sensor.**

In this study, the environmental samples were collected near mining tailings, and as the metal composition of each sample was unknown beforehand, we could not control the concentration ranges of Pb and Cu in these samples. This resulted in some limited data points, particularly for Pb, which appeared in significant concentration in only one sample. These initial tests, however, serve as an exploratory complement to the main focus of our study, which centers on sensor construction and optimization. We agree that further analysis with a greater number of environmental samples would better encompass the sensor’s detection range and precision, and we have noted this as an area for future work.

5. **The paper does not provide sufficient evidence or rationale for how and to what extent Nafion (Nf) and AgNPs enhance the sensor’s performance. For example, in lines 365-367, it is claimed that the high surface area of AgNPs contributes to the sensor’s performance. However, this assertion requires experimental validation, such as varying the diameter of AgNPs and testing their corresponding sensing performance. Similarly, the role of Nf should be experimentally examined—at the very least, by comparing the performance of AgNPs/Nf/FTO with AgNPs/FTO to demonstrate that Nf indeed improves the sensor’s performance.**

Experimental details about the role of Ag nanoparticles and Nafion in the sensor’s performance have been added. For this, we conducted comparative tests among different electrodes (FTO, Nf/FTO and AgNPs/Nf/FTO). The results are presented in Figure 4.

Additionally, scanning electron microscopy (SEM) images showing the average size of the Ag nanoparticles are included using the software ImageJ2 in Results.

6. **The paper tests only a limited number of real samples and does not provide data on the sensor's performance across a range of concentrations. To strengthen the validity of the findings, it is necessary to evaluate the sensor's performance with a broader set of real samples and across various concentrations.**

We appreciate your suggestion regarding the comparison with ICP-MS and recognize the need for a comprehensive dataset to fully establish the DPV-AgNPs/Nf/FTO sensor's capabilities across a wider concentration range. In this study, however, our primary focus was on the construction, optimization, and evaluation of analytical parameters, such as accumulation time, deposition potential, and pH, that enhance the sensor's performance. To that end, we conducted preliminary testing on real samples to provide an initial indication of the sensor’s applicability in real-world matrices. While these tests included limited concentration points, they served as a complementary demonstration rather than as exhaustive validation. We agree that a more extensive evaluation, including testing at lower concentration regimes, would provide a fuller assessment of the sensor’s performance compared to ICP-MS. Consequently, we have added this point as a recommendation for future work, noting the importance of further real sample analysis to more robustly validate the sensor's range and precision.

7. **The section on the simultaneous determination of Pb(II) and Cu(II) ions lacks sufficient detail on how the signals of Pb and Cu are de-convoluted. It is essential to include a detailed explanation of the de-convolution process. Additionally, experiments involving mixtures with varying concentrations of Pb(II) and Cu(II) ions should be conducted to test the effectiveness of your algorithm.**

To better illustrate the signals of the analytes in question, we have added graphs of the Pb and Cu peaks separately. Specific reduction and oxidation potentials were also included in the discussion using the Ag/AgCl reference electrode.

8. **Authors have reported that sensor is selective and sensitive for Pb (II) and Cu (II). However, no other heavy elements are analyzed which poses a question on the selectivity of the sensor. Moreover, a selective sensor will have high response to one analyte than the others investigated so claiming sensor selectivity for Pb(II) and Cu (II) needs an explanation. Please explain it.**

The results provided by ICP-MS reveal the presence of additional metal ions in the problem samples, with some cases showing concentrations higher than those of Pb(II) and Cu(II) ions. The low error percentages achieved between the reference concentration determined by ICP-MS and the concentration obtained using our sensor demonstrate the selectivity of our sensor for the previously mentioned analytes. To clarify this, the results of the total concentration determination provided by ICP-MS have been included as supplementary material, along with the corresponding explanation incorporated into the text.

**Responses to Specific Text Observations**

**Abstract**:

To enhance the clarity of the text, the abstract was paraphrased following the reviewers' recommendations. Additionally, the keywords were also updated accordingly.

**Introduction**:

We have included an analysis of additional techniques for heavy metal detection, comparing their effectiveness and cost with our proposed sensor. Additionally, we mentioned the concentration levels of Pb and Cu that pose a health risk.

**Methods and Materials**:

Formatting details were corrected, and relevant references were added in the analysis section.

**Results**:

Changes were made to the text format. Additionally, the size of the silver nanoparticles deposited on our electrode was included. This analysis was performed using the ImageJ2 software, and the results obtained for the protocol used were consistent with the available literature.

Similarly, the graphs were renumbered to improve their clarity and understanding.

We hope these additional modifications meet the requirements. We remain attentive to any other suggestions you may have and thank you again for your recommendations.

Sincerely,
